# Effect of patient-related factors on hospitalization service satisfaction and recommendation intention of medical institutions in Korea

**DOI:** 10.1186/s12913-023-09754-4

**Published:** 2023-06-30

**Authors:** Jeong Woo Shin, Bo Ram Choi

**Affiliations:** 1 Korea Institute for Health and Social Affairs, 370 Sicheong-Daero, Sejong, South Korea; 2 Department of Health Administration, Hanyang Cyber University, 220 Wangsimni-Ro, Seongdong-Gu, Seoul, South Korea

**Keywords:** Patient, Medical service, Satisfaction, Recommendation intention

## Abstract

**Objective:**

This study examined the factors that influence the 'Overall Satisfaction' and 'Intention to Recommend' of medical institutions used using the Korea Medical Service Experience Survey (2019–2021).

**Data sources:**

This study used the data of Medical Service Experience Survey in Korea. The data collected for data analysis were from 2019 to 2021 (Medical service period: 2018.07.01. ~ 2021.06.30).

**Study design:**

The 2019 Medical Service Experience Survey was conducted from July 8 to September 20, 2019, and a total of 12,507 people (Medical service period: 2018.07.01. ~ 2019.06.30) were collected. The 2020 survey was conducted from July 13 to October 9, 2020, and a total of 12,133 people (Medical service period: 2019.07.01 ~ 2020.06.30.) were collected. The 2021 survey was conducted from July 19 to September 17, 2021), and a total of 13,547 people were collected (Medical service period: 2020.07.01. ~ 2021.06.30). Overall satisfaction and recommendation intentions for medical institutions consist of a Likert 5-point scale. At this time, the Top-box rating model used in the United States was applied.

**Data collections/extraction methods:**

In this study, only those who used inpatient services (15 years of age or older) were included because they spent a long time in a medical institution and had an intensive experience, and a total of 1,105 subjects were included in the analysis.

**Principal findings:**

Self-rated health and the type of bed influenced overall satisfaction with medical institutions. In addition, the type of economic activity, living area, self-rated health, the type of bed, and the type of nursing service affected the intention to recommend. And it was confirmed that overall satisfaction with medical institutions and intention to recommend them were higher in the 2021 survey than in 2019.

**Conclusions:**

These results suggest that government policy on resources and systems is important. Through the case of Korea, it was found that the policy of reducing multi-person beds and expansion of integrated nursing service had a significant impact on patients' experience of using medical institutions and improving the quality of care.

## Introduction

The patient satisfaction emerged around 1980, as part of realizing customer-centered values in the health care sector worldwide. Until the 1980s, the medical field was dominated by doctors' specialized knowledge and authority, and there was a lack of interest in the emotions and psychology of patients. However, as hospital management professionals emerged in the United States in 1980, customer satisfaction, which is considered a core value in the general service industry, has also established itself in the medical industry [1]. In addition, Donabedian presented the concepts of structure, process, and performance for medical quality evaluation and suggested various methods to measure it [2], which established a "patient satisfaction survey" that subjectively evaluates patients' medical use services.

There are various factors that affect the satisfaction of patients using medical institutions. In general, medical technology for treatment and surgery, communication with medical personnel, and medical institution facilities are representative. On the other hand, patients who are satisfied with the service will be highly loyal to the medical institution, revisit the same medical institution, and recommend hospitals to others [3].

The most fundamental thing that can increase patient satisfaction is to provide patient-centered medical services. The Institute of Medicine (IOM) defines Patient-Centeredness as “a partnership between physicians and patients to ensure that decisions respect patients' wants, needs, and preferences and that patients are adequately educated and supported to make decisions and participate in their own care” [4, 5]. And the Agency for Healthcare Research and Quality (AHRQ) in the United States measures patient experience in a standardized framework from 1995 through the Consumer Assessment of Healthcare Providers and Systems (CAHPS) program (https://www.cms.gov/research-statistics-data-and-systems/research/cahps). The United States is currently using CAHPS surveys for pay-for-performance programs [6]. Specifically, the Hospital Consumer Assessment of Healthcare Providers and Systems (HCAHPS) survey is a tool to measure patients' perceptions of the hospital experience [7] and the survey results are regularly published on hospital comparison websites (http://www.medicare.gov/hospitalcompare/search.aspx). Through this, it is helping patient to choose a hospital, and medical service satisfaction surveys and evaluations have become very important socially. Furthermore, when patients are satisfied with the quality of medical services they have experienced, it carries the meaning that it can lead to positive word-of-mouth recommendations among acquaintances, which can be considered as acts of customer loyalty [8].

In line with this trend, each medical institution is naturally increasing its efforts to measure patient experience [9]. And they are trying to implement person-centered care to improve patient experiences, health outcomes, efficacy and quality of care [10, 11]. For example, patient-reported experience measures are being used for regulation and/or certification in Canada, the Czech Republic, Denmark, France, and the United Kingdom [12–14]. And the US uses this to determine the reimbursement of value-based payment systems [12, 15, 16]. Meanwhile, the OECD has recommended member countries to collect patient experience information. Since 2013, the OECD has included patient experience indicators in OECD Health Statistics, and most of countries have submitted patient experience survey data (https://stats.oecd.org/Index.aspx?ThemeTreeId=9). This movement of the international community became the basis for strengthening patient participation in the provision of medical services and ensuring the quality of medical care. Along with international discussions, the Ministry of Health and Welfare of Korea recognized the importance of confirming patient experiences and introduced a medical service experience survey for all citizens in 2017 [17]. And, the evaluation results of HCAHPS, which are frequently cited in discussions about patient experience, are published on the website. It helps patient choose hospitals by presenting comparative values such as the most positive response rate (response with 9–10 points) (http://www.medicare.gov/hospitalcompare/search.aspx) [18], is called Top-box. Although the scale and level to measure satisfaction will vary, in the United States, the Top-box value is determined to be important for patient to choose hospitals.

This study intends to examine changes in overall satisfaction and recommendation intentions, which are outcome indicators that are expected to influence patients' choice of medical institutions. To this end, we would like to analyze the experience of medical services using raw data from 2019 to 2021, the most recent data among the medical service experience surveys in Korea, based on the Top-box model. The analysis results will be used as basic data for establishing patient-centered policies, contributing to provision of better medical services to patients.

## Methods

### Study population and data collection

This study used the data of Medical Service Experience Survey in Korea. This survey is a national survey conducted annually by the Ministry of Health and Welfare and the Korea Institute for Health and Social Affairs since 2017. The survey sample was 6000 households extracted by the National Statistical Office's Population and Housing Census [19], and was sampled by the probability proportional system extraction method [20]. The raw data were obtained from the MDIS (Microdata Integrated Service) website (https://mdis.kostat.go.kr/index.do) of the National Statistical Office, and the data collected for data analysis were from 2019 to 2021.

The 2019 Medical Service Experience Survey was conducted from July 8 to September 20, 2019, and a total of 12,507 people (Medical service period: 2018.07.01. ~ 2019.06.30) were collected. The 2020 survey was conducted from July 13 to October 9, 2020, and a total of 12,133 people (Medical service period: 2019.07.01 ~ 2020.06.30.) were collected. The 2021 survey was conducted from July 19 to September 17, 2021), and a total of 13,547 people were collected. (Medical service period: 2020.07.01. ~ 2021.06.30).

**Fig. 1:**
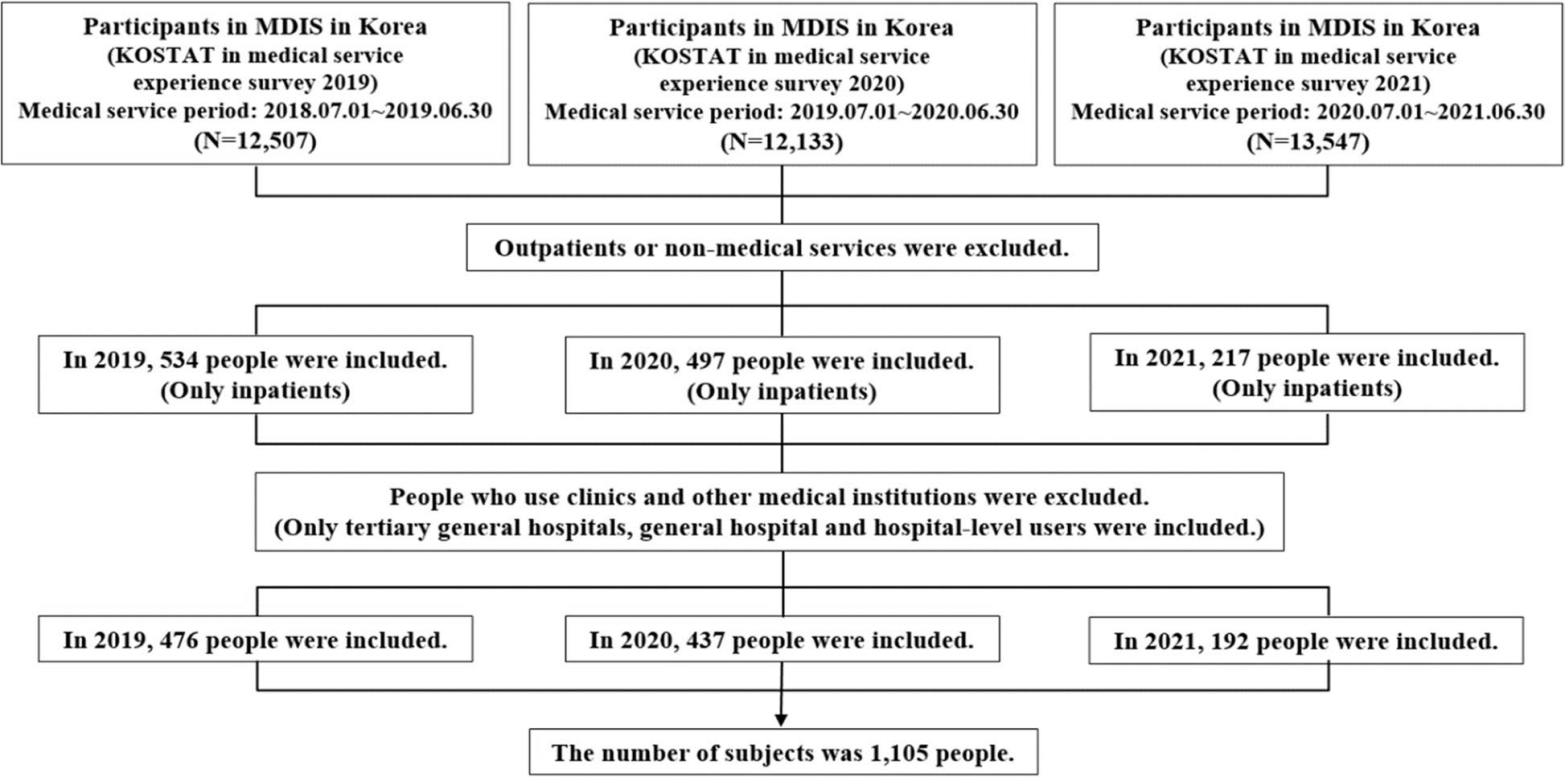
Study Flow Diagram

In this study, only those who used inpatient services (15 years of age or older) were included because they spent a long time in a medical institution and had an intensive experience. The inclusion and exclusion criteria were established according to the following procedure, and a total of 1,105 subjects were included in the analysis (Fig. 1).

### Patient-related factors

Patient's demographic factors included sex, age, education, type of economy activity, medical insurance, household income level (Q1–Q5, Q1 has a low income level and Q5 has a high income level), and living area. In case household income, it is determined by the distribution of those surveyed. However, the household balance index was applied to reflect the size of the number of household members of the household. Patient's health factors included the subjective health status (self-rated health) and the presence or absence of chronic disease Subjective health status is the health result of self-determination on a 5-point scale. The presence or absence of chronic diseases refers to whether there was one or more of the 12 diseases presented in the questionnaire such as high blood pressure and diabetes.

Structural factors for patient use of hospital are determined by the patient's intention at the time of hospitalization, corresponding to the type of hospital room (single room, double room, triple room, and multi-person room) and the type of nursing service. There are three main types of nursing services in Korea: employment of personal caregivers, family care, and integrated nursing care services. As the size of the family became smaller and the burden of caring increased, an integrated nursing care service was introduced to enable hospitalization without personal caregivers [21–23]. Since the government subsidy pilot project (2013) [24], health insurance has been applied to the entire country now, so anyone can use it with a small co-payment.

### Outcome indicators

Overall satisfaction and recommendation intentions for medical institutions consist of a Likert 5-point scale. And it means that the higher the score, the higher the overall satisfaction with the medical institution and the higher the willingness to recommend medical institution.

### Applications of HCAHPS Responses

This study analyzed the change in the percentage of those who responded most positively (response with very much) by applying the HCAHPS analysis method to subjects who used inpatient services and were discharged. The HCAHPS questionnaire consisted of 10 options and was categorized into four (Definitely no, Probably no, Probably yes, Definitely yes) [18]. Through this HCAHPS Survey, it is possible to measure and compare how patients perceive treatment in hospitals [25–27]. The Centers for Medicare and Medicaid Services (CMS) in the US analyzes the survey results in the form of a top-box (the most positive survey response) and uses them to determine hospitals’ financial bonuses or penalties [25].

Meanwhile, the Korean medical service experience survey consisted of a Likert 5-point scale, Subjects who responded with a score of 5 (response with strongly agree) were classified as a Top-box (the most positive survey response), and subjects who responded with a score of 1 to 4 were classified as a Non-top-box. That is, it was analyzed by coding 1 (Top-box) and 0 (Non-top-box) according to the high level of satisfaction.

### Statistical analysis

For the collected data, IBM SPSS 25.0 version program was used. First, Frequency analysis was conducted to find out the socio-demographic characteristics of the study subjects who used inpatient services. Second, the changes in overall satisfaction and recommendation intention according to the study subjects' patient-related factors (Patient's demographic factors, Patient's health factors and Patient's health factors) and the period of medical service use were compared and analyzed through cross-analysis. Third, logistic regression analysis was performed using these as dependent variables to analyze the factors affecting overall satisfaction and recommendation intention.

## Results

### General characteristics of the study subjects

The general characteristics of the study subjects are shown in Table 1. A total of 1,105 subjects were 476 (43.1%) who used inpatient services in the first period (2018.07.01. ~ 2019.06.30.), 439 (39.5%) who used them in the second period (2019.07.01. ~ 2020.06.30.), and 192 (17.4%) who used them in the third period (2020.07.01. ~ 2021.06.30.). According to the three-year data, the population aged 60 or older accounted for the largest portion with 664 (60.1%), and the number of chronically ill patients accounted for about two-thirds with 712. And the proportion of users of the integrated nursing care service showed an increasing trend every year.

**Table 1:** General characteristics of study subjects

Variables			Survey 2019 (2018.07.01. ~ 2019.06.30.)	Survey 2020 (2019.07.01. ~ 2020.06.30.)	Survey 2021 (2020.07.01. ~ 2021.06.30.)	Total
Patient's demographic factors	Sex	Men	208(43.7)	188(43.0)	76(39.6)	472(42.7)
		Women	268(56.3)	249(57.0)	116(60.4)	633(57.3)
	Age	15 ~ 39	49(10.3)	39(8.9)	33(17.2)	121(11.0)
		40 ~ 49	49(10.3)	58(13.3)	11(5.7)	118(10.7)
		50 ~ 59	84(17.6)	83(19.0)	35(18.2)	202(18.3)
		over 60	294(61.8)	257(58.8)	113(58.9)	664(60.1)
	Education	Below primary	139(29.2)	122(27.9)	37(19.3)	298(27.0)
		Secondary	253(53.2)	223(51.0)	111(57.8)	587(53.1)
		Higher	84(17.6)	92(21.1)	44(22.9)	220(19.9)
	Type of economy activity	Wage worker	84(17.6)	91(20.8)	44(22.9)	219(19.8)
		Self-employed & employers	76(16.0)	91(20.8)	41(21.4)	208(18.8)
		Housewives	156(32.8)	146(33.4)	65(33.9)	367(33.2)
		Student & Others	160(33.6)	109(24.9)	42(21.9)	311(28.1)
	Medical insurance	National Health Insurance	441(92.6)	416(95.2)	179(93.2)	1,036(93.8)
		Medical aid program	35(7.4)	21(4.8)	13(6.8)	69(6.2)
	Household income level	1Q	153(32.1)	140(32.0)	79(41.1)	372(33.7)
		2Q	118(24.8)	102(23.3)	33(17.2)	253(22.9)
		3Q	58(12.2)	79(18.1)	22(11.5)	159(14.4)
		4Q	77(16.2)	52(11.9)	25(13.0)	154(13.9)
		5Q	70(14.7)	64(14.6)	33(17.2)	167(15.1)
	Living area	Urban	321(67.4)	289(66.1)	124(64.6)	734(66.4)
		Rural	155(32.6)	148(33.9)	68(35.4)	371(33.6)
Patient's health factors	Subjective health status	Very poor	42(8.8)	32(7.3)	10(5.2)	84(7.6)
		Poor	143(30.0)	148(33.9)	56(29.2)	347(31.4)
		Moderate	152(31.9)	138(31.6)	61(31.8)	351(31.8)
		Good	126(26.5)	95(21.7)	54(28.1)	275(24.9)
		Very good	13(2.7)	24(5.5)	11(5.7)	48(4.3)
	Chronic diseases	No	170(35.7)	148(33.9)	75(39.1)	393(35.6)
		Yes	306(63.4)	289(66.1)	117(60.9)	712(64.4)
Structural factors for patient use of hospital	Type of room	Single room	19(4.0)	17(3.9)	7(3.6)	43(3.9)
		Double room	96(20.2)	66(15.1)	39(20.3)	201(18.2)
		Triple room	18(3.8)	21(4.8)	11(5.7)	50(4.5)
		Multi-person room	343(72.1)	333(76.2)	135(70.3)	811(73.4)
	Type of nursing service	No	119(25.0)	122(27.9)	49(25.5)	290(26.2)
		Integrated nursing care services	51(10.7)	73(16.7)	39(20.3)	163(14.8)
		Family care	254(53.4)	209(47.8)	84(43.8)	547(49.5)
		Employment of personal caregivers	52(10.9)	33(7.6)	20(10.4)	105(9.5)
Total			476(43.1)	437(39.5)	192(17.4)	1,105(100.0)

### Top-box rating and Non-top-box rating of overall satisfaction

Table 2 shows the results of analyzing the ratio of Top-box rating and Non-top-box rating of overall satisfaction among patient-related factors. As for overall satisfaction, the Top-box rating was 137 (12.4%) and the Non-top-box rating was 968 (87.6%). On the other hand, in terms of overall satisfaction, the Top-box ratings differed according to the subject's education level, self-rated health, and period of medical service use.

**Table 2:** Top box rating of overall satisfaction according to patient-related factors

Variables			Non top-box (1–4) *n* = 968	Top-box (5) *n* = 137	χ^2^	*p* value
Patient's demographic factors	Sex	Men	413(87.5)	59(12.5)	0.008	0.929
		Women	555(87.7)	78(12.3)		
	Age	15 ~ 39	102(84.3)	19(15.7)	2.707	0.439
		40 ~ 49	100(84.7)	18(15.3)		
		50 ~ 59	178(88.1)	24(11.9)		
		over 60	588(88.6)	76(11.4)		
	Education	Below primary	262(87.9)	36(12.1)	7.602	0.022
		Secondary	525(89.4)	62(10.6)		
		Higher	181(82.3)	39(17.7)		
	Type of economy activity	Wage worker	194(88.6)	25(11.4)	0.667	0.881
		Self-employed and employers	179(86.1)	29(13.9)		
		Housewives	322(87.7)	45(12.3)		
		Student & Others	273(87.8)	38(12.2)		
	Medical insurance	National Health Insurance	907(87.5)	129(12.5)	0.044	0.834
		Medical aid program	61(88.4)	8(11.6)		
	Household income level	1Q	321(86.3)	51(13.7)	7.154	0.128
		2Q	232(91.7)	21(8.3)		
		3Q	135(84.9)	24(15.1)		
		4Q	138(89.6)	16(10.4)		
		5Q	142(85.0)	25(15.0)		
	Living area	Urban	645(87.9)	89(12.1)	1.150	0.699
		Rural	323(87.1)	48(12.9)		
Patient's health factors	Subjective health status	Very poor	75(89.3)	9(10.7)	34.645	< 0.001
		Poor	310(89.3)	37(10.7)		
		Moderate	313(89.2)	38(10.8)		
		Good	241(87.6)	34(12.4)		
		Very good	29(60.4)	19(39.6)		
	Chronic diseases	No	340(86.5)	53(13.5)	0.665	0.415
		Yes	628(88.2)	84(11.8)		
Structural factors for patient use of hospital	Type of room	Single room	31(72.1)	12(27.9)	11.695	0.009
		Double room	175(87.1)	26(12.9)		
		Triple room	47(94.0)	3(6.0)		
		Multi-person room	715(88.2)	96(11.8)		
	Type of nursing service	No	253(87.2)	37(12.8)	0.950	0.813
		Integrated nursing care services	140(85.9)	23(14.1)		
		Family care	484(88.5)	63(11.5)		
		Employment of personal caregivers	91(86.7)	14(13.3)		
Period of use of medical services		2018.07.01. ~ 2019.06.30	413(86.8)	63(13.2)	9.528	0.009
		2019.07.01. ~ 2020.06.30	397(90.8)	40(9.2)		
		2020.07.01. ~ 2021.06.30	158(82.3)	34(17.7)		
Total			968(87.6)	137(12.4)	-	-

### Top-box rating and Non-top-box rating of recommendation intentions.

Table 3 shows the results of analyzing the ratio of Top-box rating and Non-top-box rating of recommendation intentions. The intention to recommend medical institutions was 159 (14.4%) with Top-box rating and 946 (85.6%) with Non-top-box rating. Top-box ratings differed according to the subject's living area, self-rated health, and period of medical service use.

**Table 3:** Top box rating of recommendation intention according to patient-related factors

Variables			Non top-box (1–4) *n* = 946	Top-box (5) *n* = 159	χ^2^	*p* value
Patient's demographic factors	Sex	Men	413(87.5)	59(12.5)	2.387	0.122
		Women	533(84.2)	100(15.8)		
	Age	15 ~ 39	102(84.3)	19(15.7)	3.928	0.269
		40 ~ 49	103(87.3)	15(12.7)		
		50 ~ 59	181(89.6)	21(10.4)		
		over 60	560(84.3)	104(15.7)		
	Education	Below primary	247(82.9)	51(17.1)	3.102	0.212
		Secondary	512(87.2)	75(12.8)		
		Higher	187(85.0)	33(15.0)		
	Type of economy activity	Wage worker	198(90.4)	21(9.6)	5.124	0.163
		Self-employed and employers	176(84.6)	32(15.4)		
		Housewives	310(84.5)	57(15.5)		
		Student & Others	262(84.2)	49(15.8)		
	Medical insurance	National Health Insurance	883(85.2)	153(14.8)	1.937	0.164
		Medical aid program	63(91.3)	6(8.7)		
	Household income level	1Q	314(84.4)	58(15.6)	3.957	0.412
		2Q	210(83.0)	43(17.0)		
		3Q	139(87.4)	20(12.6)		
		4Q	136(88.3)	18(11.7)		
		5Q	147(88.0)	20(12.0)		
	Living area	Urban	664(87.7)	90(12.3)	8.033	0.005
		Rural	302(81.4)	69(18.6)		
Patient's health factors	Subjective health status	Very poor	72(85.7)	12(14.3)	17.175	0.002
		Poor	292(84.1)	55(15.9)		
		Moderate	309(88.0)	42(12.0)		
		Good	241(87.6)	34(12.4)		
		Very good	32(66.7)	16(33.3)		
	Chronic diseases	No	342(87.0)	51(13.0)	0.987	0.320
		Yes	604(84.8)	108(15.2)		
Structural factors for patient use of hospital	Type of room	Single room	33(76.7)	10(23.3)	4.392	0.222
		Double room	174(86.6)	27(13.4)		
		Triple room	40(80.0)	10(20.0)		
		Multi-person room	699(86.2)	112(13.8)		
	Type of nursing service	No	255(87.9)	35(12.1)	18.572	< 0.001
		Integrated nursing care services	122(74.8)	41(25.2)		
		Family care	475(86.8)	72(13.2)		
		Employment of personal caregivers	94(89.5)	11(10.5)		
Period of use of medical services		2018.07.01. ~ 2019.06.30	439(92.2)	37(7.8)	32.494	< 0.001
		2019.07.01. ~ 2020.06.30	359(82.2)	78(17.8)		
		2020.07.01. ~ 2021.06.30	148(77.1)	44(22.9)		
Total			946(85.6)	159(14.4)	-	-

### Results of logistic regression analysis for overall satisfaction

The results of logistic regression analysis for overall satisfaction are shown in Table 4. Among the patient's health factors, the more positively the health status was judged, the more likely it was to give a Top-box grade in overall satisfaction. Those who thought they were very good compared to those who thought their health was very poor (OR = 8.927, 95% CI = 3.077 to 25.896) were likely to grant the Top-box ratings. Among the structural factors for patients' hospital use, the type of hospital room was statistically significantly less Top-box rating when admitted to a double room (OR = 0.400, 95% CI = 0.173 to 0.923), a triple room (OR = 0.202, 95% CI = 0.050 to 0.815), and multi-person (OR = 0.389, 95% CI = 0.183 to 0.828) than a single room. In addition, the patient's medical service use period was less likely to give a Top-box rating to the second period (OR = 0.537, 95% CI = 0.342 to 0.843) compared to the first period.

**Table 4:** Logistic regression analysis of the effect of patient-related factors on overall satisfaction

Variables			B	SE	OR	95% CI	*p* value
Patient's demographic factors	Sex	Men			1.000		
		Women	-0.110	0.248	0.896	0.551–1.457	0.658
	Age	15 ~ 39			1.000		
		40 ~ 49	0.571	0.409	1.769	0.794–3.945	0.163
		50 ~ 59	0.624	0.412	1.867	0.833–4.183	0.129
		over 60	0.457	0.422	1.579	0.691–3.609	0.278
	Education	Below primary			1.000		
		Secondary	-0.152	0.274	0.859	0.502–1.468	0.577
		Higher	0.560	0.396	1.750	0.805–3.804	0.158
	Type of economy activity	Wage worker			1.000		
		Self-employed and employers	0.444	0.327	1.559	0.822–2.958	0.174
		Housewives	0.362	0.348	1.436	0.726–2.839	0.298
		Student & Others	0.400	0.341	1.491	0.764–2.911	0.241
	Medical insurance	National Health Insurance			1.000		
		Medical aid program	-0.103	0.421	0.902	0.395–2.059	0.807
	Household income level	1Q			1.000		
		2Q	-0.602	0.305	0.547	0.301–0.996	0.048
		3Q	0.153	0.315	1.165	0.628–2.160	0.628
		4Q	-0.654	0.367	0.520	0.253–1.068	0.075
		5Q	-0.222	0.338	0.801	0.413–1.554	0.512
	Living area	Urban			1.000		
		Rural	0.069	0.211	1.072	0.709–1.620	0.743
Patient's health factors	Subjective health status	Very poor			1.000		
		Poor	0.065	0.408	1.067	0.480–2.374	0.873
		Moderate	0.158	0.425	1.171	0.509–2.693	0.710
		Good	0.466	0.449	1.593	0.661–3.837	0.299
		Very good	2.189	0.543	8.927	3.077–25.896	< 0.001
	Chronic diseases	No			1.000		
		Yes	0.267	0.263	1.306	0.780–2.188	0.310
Structural factors for patient use of hospital	Type of room	Single room			1.000		
		Double room	-0.917	0.427	0.400	0.173–0.923	0.032
		Triple room	-1.600	0.712	0.202	0.050–0.815	0.025
		Multi-person room	-0.943	0.385	0.389	0.183–0.828	0.014
	Type of nursing service	No			1.000		
		Integrated nursing care services	0.366	0.308	1.442	0.788–2.638	0.235
		Family care	-0.119	0.241	0.888	0.553–1.425	0.623
		Employment of personal caregivers	0.164	0.365	1.179	0.576–2.410	0.653
Period of use of medical services		2018.07.01. ~ 2019.06.30			1.000		
		2019.07.01. ~ 2020.06.30	-0.622	0.230	0.537	0.342–0.843	0.007
		2020.07.01. ~ 2021.06.30	0.250	0.249	1.284	0.789–2.090	0.315

### Results of logistic regression analysis on the intention to recommend medical institutions

The results of logistic regression analysis on the intention to recommend medical institutions are shown in Table 5. Among patient's health factors, in the case of type of economy activity, students and others (OR = 2.508, 95% CI = 1.250–5.031) was more likely to give a Top-box rating than wage worker. The person living in rural area (OR = 1.627, 95% CI = 1.090 to 2.429) was more likely to give a Top-box rating rather than living in urban area. Compared to those who did not use the nursing service, those who used the nursing care integrated service (OR = 2.469, 95% CI = 1.407 to 4.333) were more likely to give the Top-box rating. And the duration of patient's use of medical services was likely to give Top-box rates in the second period (OR = 3.144, 95% CI = 1.980 to 4.991) and the third period (OR = 3.976, 95% CI = 2.321 to 6.810), compared to the first period.

**Table 5:** Logistic regression analysis of the effect of patient-related factors on recommendation intention

Variables			B	SE	OR	95% CI	*p* value
Patient's demographic factors	Sex	Men			1.000		
		Women	0.316	0.233	1.372	0.852–2.210	0.194
	Age	15 ~ 39			1.000		
		40 ~ 49	0.262	0.440	1.300	0.549–3.079	0.551
		50 ~ 59	0.022	0.435	1.022	0.436–2.400	0.959
		over 60	0.258	0.428	1.295	0.559–2.998	0.546
	Education	Below primary			1.000		
		Secondary	-0.164	0.259	0.848	0.510–1.411	0.526
		Higher	0.306	0.401	1.357	0.619–2.976	0.446
	Type of economy activity	Wage worker			1.000		
		Self-employed and employers	0.616	0.351	1.851	0.930–3.684	0.080
		Housewives	0.555	0.353	1.742	0.872–3.478	0.116
		Student & Others	0.919	0.355	2.508	1.250–5.031	0.010
	Medical insurance	National Health Insurance			1.000		
		Medical aid program	-0.726	0.489	0.484	0.186–1.261	0.138
	Household income level	1Q			1.000		
		2Q	0.496	0.265	1.641	0.977–2.758	0.061
		3Q	-0.077	0.339	0.926	0.476–1.799	0.820
		4Q	-0.020	0.357	0.980	0.487–1.972	0.954
		5Q	-0.156	0.364	0.856	0.427–1.673	0.669
	Living area	Urban			1.000		
		Rural	0.487	0.204	1.627	1.090–2.429	0.017
Patient's health factors	Subjective health status	Very poor			1.000		
		Poor	-0.014	0.376	0.986	0.472–2.059	0.971
		Moderate	-0.127	0.397	0.880	0.404–1.917	0.778
		Good	0.056	0.427	1.058	0.458–2.443	0.754
		Very good	0.931	0.546	2.537	0.871–7.394	0.088
	Chronic diseases	No			1.000		
		Yes	0.324	0.260	1.383	0.831–2.300	0.212
Structural factors for patient use of hospital	Type of room	Single room			1.000		
		Double room	-0.352	0.479	0.704	0.275–1.798	0.463
		Triple room	0.333	0.574	1.395	0.453–4.297	0.562
		Multi-person room	-0.589	0.443	0.555	0.233–1.321	0.183
	Type of nursing service	No			1.000		
		Integrated nursing care services	0.904	0.287	2.469	1.407–4.333	0.002
		Family care	0.166	0.248	1.180	0.726–1.917	0.504
		Employment of personal caregivers	-0.301	0.400	0.740	0.338–1.620	0.452
Period of use of medical services		2018.07.01. ~ 2019.06.30			1.000		
		2019.07.01. ~ 2020.06.30	1.145	0.236	3.144	1.980–4.991	< 0.001
		2020.07.01. ~ 2021.06.30	1.380	0.275	3.976	2.321–6.810	< 0.001
Overall satisfaction		Non top-box (1–4)			1.000		
		Top-box (5)	1.641	0.234	5.161	3.261–8.167	< 0.001

Furthermore, the correlation coefficient between the recommendation intentions and overall satisfaction was 0.237 (*p <* 0.000), indicating that as overall satisfaction increased, the recommendation intentions also increased. And the group with high overall satisfaction (Top-box rating) (OR = 5.161, 95% CI = 3.261 to 8.167) was more likely to give a Top-box rating about recommendation intention rather than Non top-box rating.

## Discussion

According to the distribution of subjects in this study, there were fewer inpatients in 2021 compared to the 2019 and 2020 surveys. In 2021, the number of hospitalized patients is estimated to decrease due to COVID-19. Meanwhile, over the past three years, those in their 60 s or older accounted for more than 60% of inpatients, and those with chronic diseases accounted for more than 60%, confirming the need for medical care of the vulnerable.

According to the analysis results, the recommendation intention and overall satisfaction with the medical institution corresponding to the patient-related factors showed a high level of satisfaction (Top-box rating) for those who judged their health to be good and those who used single-person rooms. And participants who responded positively to their subjective health status rated the overall satisfaction with the medical institution higher. This suggests that individuals who have a positive perception of their health status tend to evaluate the quality of medical services more positively [28]. On the other hand, it is thought that the single-person room would lead to a positive experience of inpatients in terms of less noise in environmental aspects and protection of personal privacy. As for the intention to recommend the medical institution used, the non-wage workers, those living in rural are, those who thought their health was good, and those who used the single-person room showed a high level of satisfaction (Top-box rating).

In addition, those who used the integrated nursing care service showed a higher level of satisfaction and recommendation intention (Top-box rating) than those who did not use the nursing service. In Korea, the integrated nursing care service is innovative change. Until then, households suffered mental and economic burdens by caring for their families or hiring personal caregiver privately. In particular, most of those who hired a personal caregiver complained of a high burden on cost [29]. Among these, the newly settled integrated nursing care service system was a good opportunity to increase patient satisfaction in terms of both time and cost.

In both patient satisfaction and recommendation intention, patients admitted to single rooms had higher scores than multi-person rooms. Several previous studies have also shown that single-person rooms are superior in both patient satisfaction and quality of care compared to multi-person rooms [30–32]. In Korea, patients prefer single rooms to multi-person rooms [33, 34], but in terms of bed composition and price burden, they are practically hospitalized in multi-person rooms. This is because hospital beds are operated mainly in multi-person rooms. In addition, since single-room room fees are not covered by health insurance, the patient must directly pay the entire hospital room fee unless there is a special reason (infectious disease and deathbed, etc.). The government and medical institutions should come up with bed policies that reflect consumers' preferences, such as reducing multi-person rooms and lowering the cost of single rooms.

Overall satisfaction with medical institutions and the intention to recommend them are representative performance indicators for medical institutions, and are very important items to be checked by medical institutions. In this study, overall satisfaction and recommendation intention were higher in the 2021 survey than in the 2019 survey. The reason for this increase in satisfaction may be the result of quality interventions based on previously measured results [35]. This can be seen as the result of medical institutions' efforts to create a patient-centered medical culture and provide medical services from the perspective of patients despite the risk of COVID-19. Of course, there should be long-term review and microscopic confirmation, but the government's institutional intervention, such as the expansion of patient experience evaluation (Government-led, starting from 2017), also contributed to the efforts of medical institutions.

The main efforts to increase satisfaction with the use of medical services are made by individual medical institutions, but it is the role of the government to create such an environment. This is why the World Health Organization (WHO) has suggested that improving responsiveness is a major goal of the health care system [36]. It is important to have an institutional infrastructure so that patients can have a good experience even in the non-medical aspect. The government should establish stewardship of health and medical policies, such as presenting a desirable image of medical provision. When the government and medical institutions work together, medical quality improvement activities can create synergy. When the government and medical institutions work together, health care quality improvement activities can create synergy.

On the other hand, this study has some limitations. First, the basic data of this analysis are mainly characteristics of patients, and the influence of characteristics of medical providers such as the size of medical institutions and medical subjects on patients was not included in the analysis. In particular, physical resources such as facilities and medical personnel, along with the size of medical institutions, can have a significant impact on the patient's experience, but this study did not reflect this. This is because this survey does not collect information from medical institutions. There is a tertiary hospital in Korea, which has modern facilities to meet the needs of various patients. It is regrettable that there is no such information in the questionnaire.

Second, from the consumer's point of view, the experience may vary depending on the initial level of expectation for the medical institution that was not reflected due to the lack of survey questions. In addition, it is difficult to judge the value of experience in a medical institution only by the area where the patient lives. Because it is easy to move to other regions due to the nature of Korea, patients often move to large cities including the Seoul metropolitan area for medical treatment unless it is a mild disease. Therefore, not only the area where the patient lives but also the location information of the medical institution must be presented to prove the validity of the conclusion that patients living in the countryside are highly satisfied. As such, there are no major variables that affect the experience, so there are constraints on interpretation.

Third, because it is a cross-sectional survey data (different subjects for each time point), not a longitudinal survey data (individual follow-up survey at intervals of time), satisfaction and recommendation intention that can be changed by environmental improvement and policy intervention could not be confirmed.

Despite the above limitations, the medical service experience survey used in this study is the only survey that can comprehensively confirm the medical experience in Korea. And with the advantage of being composed of a sample that can be representative of the whole country, it was possible to derive representative results on the factors affecting the overall satisfaction with hospitalization services and the intention to recommend medical institutions used.

## Conclusion

This study examined the changes in overall satisfaction with medical institutions and recommendation intention for inpatients using the raw data of the Medical Service Experience Survey (2019–2021) in Korea. In addition, various influencing factors were analyzed from the patient's perspective. Between July 1, 2018 and June 30, 2021, people who used inpatient services were divided into three periods, and it was confirmed that overall satisfaction with medical institutions and intention to recommend them were higher in the 2021 survey than in 2019. Overall, it can be said that this is a positive result that the provision of patient-centered medical services has been gradually activated.

In this trend, the government should expand the patient-centered system and improve the positive experience and satisfaction of medical institutions. From a policy point of view, the number of inpatients per bed should be reduced, such as reducing beds in multi-person rooms. And it should be changed to a structure in which the state is responsible for nursing and nursing together. As a result, patients will be able to concentrate on treatment with a comfortable mind, and the quality of medical care will be improved.

At the same time, it is hoped that the contents of the medical service experience survey will be expanded to build various grounds that can lead the people-centered health care system.

## Data Availability

The dataset used for analysis of this study are available for the MDIS (Microdata Integrated Service) website (https://mdis.kostat.go.kr/eng/index.do) of the National Statistical Office in KOREA. Statistics Korea not only collects microdata of self-produced statistics, but also collects microdata of other statistical agencies such as research institutes in one place, so that people can conveniently use various statistical data through MDIS.
